# Modeling space radiation induced cognitive dysfunction using targeted and non-targeted effects

**DOI:** 10.1038/s41598-021-88486-z

**Published:** 2021-04-23

**Authors:** Igor Shuryak, David J. Brenner, Steven R. Blattnig, Barbara Shukitt-Hale, Bernard M. Rabin

**Affiliations:** 1 Center for Radiological Research, Columbia University Irving Medical Center, 630 West 168th street, VC-11-234/5, New York, NY 10032 USA; 2 NASA Langley Research Center, Hampton, VA USA; 3 Human Nutrition Research Center on Aging, USDA-ARS, Tufts University, Boston, MA USA; 4 Department of Psychology, University of Maryland Baltimore County, Baltimore, MD USA

**Keywords:** Biophysics, Computational biology and bioinformatics, Neuroscience

## Abstract

Radiation-induced cognitive dysfunction is increasingly recognized as an important risk for human exploration of distant planets. Mechanistically-motivated mathematical modeling helps to interpret and quantify this phenomenon. Here we considered two general mechanisms of ionizing radiation-induced damage: targeted effects (TE), caused by traversal of cells by ionizing tracks, and non-targeted effects (NTE), caused by responses of other cells to signals released by traversed cells. We compared the performances of 18 dose response model variants based on these concepts, fitted by robust nonlinear regression to a large published data set on novel object recognition testing in rats exposed to multiple space-relevant radiation types (H, C, O, Si, Ti and Fe ions), covering wide ranges of linear energy transfer (LET) (0.22–181 keV/µm) and dose (0.001–2 Gy). The best-fitting model (based on Akaike information criterion) was an NTE + TE variant where NTE saturate at low doses (~ 0.01 Gy) and occur at all tested LETs, whereas TE depend on dose linearly with a slope that increases with LET. The importance of NTE was also found by additional analyses of the data using quantile regression and random forests. These results suggest that NTE-based radiation effects on brain function are potentially important for astronaut health and for space mission risk assessments.

## Introduction

Radiation-induced central nervous system (CNS) damage and consequent cognitive dysfunction are increasingly recognized as important risks for astronauts on long-distance space missions such as exploration of Mars^1–7^. Mechanistically-motivated mathematical modeling of this phenomenon can provide much needed insight into interpreting the growing amount of relevant experimental data in laboratory animals, generating and testing mechanistic hypotheses, and producing quantitative predictions for radiation quality effects and risk magnitudes for space mission scenarios^4,6^.

Here we developed and used several model variants based on two general categories of radiation-induced damage: targeted effects (TE), caused by traversal of cells by ionizing tracks, and non-targeted effects (NTE), caused by responses of nearby or even distant cells to signals released by traversed cells. The NTE-based terms used in these formalisms were motivated by our previous work^8–10^, where we assumed that NTE signals cause sensitive cells to enter into a prolonged stressed state (*e.g.* persistent oxidative stress) which increases the risk of adverse health effects such as carcinogenesis or cognitive dysfunction. A commonly-observed property of NTE is a non-linear concave dose response shape that increases steeply at low doses (where, for high linear energy transfer (LET) radiation exposures, not all cell nuclei are traversed by ionizing tracks) and becomes shallower or saturates at higher doses. In contrast, TE dose responses generally exhibit linear or linear-quadratic (convex) dose response shapes.

NTE-based models were previously applied to a variety of radiation damage endpoints such as carcinogenesis, cell survival, and chromosomal aberrations^8,11–18^. Although the mechanisms of radiation-induced CNS dysfunction are not yet fully understood and are being actively studied, we hypothesize that NTE may be involved in this phenomenon. Experimental evidence supporting this hypothesis includes the finding that body-only exposure to space-relevant radiation, which does not directly traverse the brain by ionizing tracks, can nevertheless affect cognitive functioning in rodents^19,20^. The molecular mechanisms of this phenomenon likely involve radiation-induced oxidative stress and neuroinflammation, which, in turn, affect neuronal function^20^. They may be mediated by blood-borne factors (*e.g.* cytokines), immune system involvement, the vagus nerve, or radiation-induced intestinal microbiome alterations^19,20^.

These findings suggest that that direct effects of HZE particles on neurons are not necessary to produce changes in neuronal function^20^. They can be viewed in a more general context, where the types of signals that propagate NTE between cells are very diverse^21–24^. Such signals include small molecules capable of moving through gap junctions (*e.g.* lipid peroxide products, inosine nucleotides), long-range signals like pro-inflammatory cytokines (*e.g.* tumor necrosis factor-α)^24^, and possibly micro RNAs^24^ and exosomes^25^. Such signaling could be involved in a variety of diseases including CNS dysfunction and carcinogenesis. The types of radiation damage that trigger NTE signal production can include unrepaired/misrepaired DNA double strand breaks, protein and lipid oxidation by radiation-induced reactive oxygen and nitrogen species, and mitochondrial damage. These events may lead to chronic inflammation, oxidative stress, and microglia activation^7^.

Based on this reasoning, we applied TE- and/or NTE-based dose response models to a large data set on novel object recognition testing in rats exposed to a variety of space-relevant radiation types and doses, published by Rabin et al*.*^26^. Our primary goals were: (1) To set up a modeling framework for evaluating combined TE and NTE mechanisms using available CNS dysfunction data. (2) To evaluate whether or not some degree of discrimination between model assumptions (*e.g.* TE vs NTE-dominant explanations for the observed radiation responses, modulated by dose and LET) could be made based on such data, and what this could imply for quantifying the risks and radiation quality dependences of this phenomenon in relation to space exploration. As more biological information about radiation effects on the CNS becomes available, more detailed hypothesis can be developed and tested using combinations of specific TE and NTE models with experimental studies.

## Materials and methods

### Data set

We selected the data set published by Rabin et al*.*^26^ for the following reasons: (1) One strain of laboratory animals (male Sprague–Dawley rats) was exposed to a broad range of space-relevant radiations (H, C, O, Si, Ti, and Fe ions), and cognitive performance was assessed using the same behavioral endpoint – novel object recognition (NOR). The NOR was chosen because it is a standard measure of cognitive performance in studies utilizing animal models. It is a measure of an organism’s ability to remember a prior interaction with a novel object. It utilizes a rat’s innate preference to interact with a novel object compared to a familiar object. This requires that the subject remember the object with which it has interacted previously. This task is routinely used to study the effects a variety of treatments on the ability of the subject to recall (remember) an object with which it has interacted previously. It was selected because there is an extensive literature available about the performance of rats following exposure to toxic treatments.

The data set came from the same institution and one NOR methodology was used throughout. This consistency should minimize the variability due to differences between animal strains/species and test types, potentially allowing the effects of radiation to be evaluated more clearly. (2) The LET range was broad, from 0.22 to 181 keV/µm, which covers most of the spectrum seen in space. (3) The dose range was also wide, including very low doses (0.001 to 0.05 Gy) as well as high doses (1–2 Gy). (4) The effects of time after exposure were assessed by performing the novel object recognition test at two time points after irradiation (1 to 17 months since exposure). The full data for each rat and study condition were kindly provided by Dr. Rabin, which we combined and processed for analysis (923 samples, Supplementary Data File online).

### Data processing

The outcome (dependent) variable in the analyzed data set was the fraction of time that a rat spent exploring the novel object^26^. Because these data are by definition fractions between 0 and 1, we log-transformed them to bring the distribution closer to normal and performed subsequent dose response modeling on the transformed scale, conceptually analogous to modeling of cell survival dose responses. In this way, we generated the outcome variable “Response” as follows, where F_nov_ is the fraction of time spent exploring the novel object reported in Rabin et al*.*^26^:

1 $$Response= -\hbox{ln}\left[{F}_{nov}\right]$$

This log-transformation changed the data scale from a fraction between 0 and 1 to a continuous number ≥ 0, which is more amenable to dose response analysis using models such as linear or linear quadratic.

### Radiation response modeling

We considered three variables – dose, LET, and time since exposure (irradiation) – as the most reasonable potential predictors of the response in this data set. To assess their relative strengths, we calculated Spearman’s correlation coefficients of each of these variables with the response variable (Response) using *R* 4.0.2 software. The correlation coefficient for time with the response was close to zero (0.00102) and not statistically significant, suggesting that time was the least important variable to consider. If time was added as a predictor in the models described below, it did not reach statistical significance, and therefore we did not include it in further analysis. To investigate the issue of radiation quality dependence, LET was binned into four categories (identified by index *i*): low (labeled L, 0.22 keV/µm), medium (labeled M, 13–16 keV/µm), high (labeled H, 41–50 keV/µm) and very high (labeled VH, 106–181 keV/µm). Radiation dose (in Gy) was treated as a continuous variable.

We modeled the radiation response using several model structures that differ in their assumptions about the dose dependences (*e.g.* linear or quadratic) and LET dependences of the TE and/or NTE components. Exploratory calculations showed poor support for complex models that included different TE (with linear and/or quadratic dependences on dose) along with different NTE coefficients for each LET category. In the fits of such models, many parameters – particularly the TE terms – had very large uncertainties. For example, the only TE term in these highly parametrized models that achieved statistical significance (*p* value = 0.0375) was the one representing a quadratic dependence on dose for the highest (VH) LET category. To reduce parameter uncertainties and clarify differences in performance between different sets of model structure assumptions, we generated 18 less parametrized simpler model variants (listed in Table 1) for more detailed evaluation. In our notation, *D* is radiation dose (in Gy), *B* is the baseline response parameter in unirradiated rats, and *kTE* and *kNTE* are parameters that represent TE and NTE, respectively. The TE or NTE parameters were allowed to differ by LET category (Table 1). In some simplified models (labeled S1 to S4), TE parameters were allowed to be adjustable only for specified LET categories (*e.g. i* = LM, H, VH indicates that TE parameters were the same for L and M LET categories, but different for H and VH categories) and/or set to zero for certain LET categories (*e.g. i* = VH indicates that TE parameters were non-zero only for the VH LET category).

**Table 1:** Comparisons of all tested model formalisms.

Model	Equation	∆AICc	R^2^	RMSE	MAE
TE__lin_	$R=B+{\sum }_{i}{kTE}_{i}\times {D}_{i}$	106.4	0.06	0.25	0.19
TE__quad_	$R=B+{\sum }_{i}{kTE}_{i}\times {D}_{i}^{2}$	80.5	0.04	0.15	0.19
NTE	$R=B+{\sum }_{i}{kNTE}_{i}\times (1-\hbox{exp}\left[-{10}^{3}\times {D}_{i}\right])$	15.9	0.12	0.24	0.18
TE__lin_noLET_	$R=B+kTE\times D$	104.8	0.04	0.25	0.19
TE__quad_noLET_	$R=B+kTE\times {D}^{2}$	102.4	0.02	0.15	0.19
NTE__noLET_	$R=B+kNTE\times (1-\hbox{exp}\left[-{10}^{3}\times D\right])$	31.8	0.09	0.24	0.18
NTE_TE__lin_noLET_	$R=B+kNTE\times (1-\hbox{exp}\left[-{10}^{3}\times D\right])+kTE\times D$	22.8	0.10	0.24	0.18
NTE_TE__quad_noLET_	$R=B+kNTE\times (1-\hbox{exp}\left[-{10}^{3}\times D\right])+kTE\times {D}^{2}$	34.3	0.10	0.24	0.18
NTE_TE__lin_	$R=B+kNTE\times (1-\hbox{exp}\left[-{10}^{3}\times D\right])+{\sum }_{i}{kTE}_{i}\times {D}_{i}$	3.8	0.11	0.24	0.18
NTE_TE__lin_S1_	$R=B+kNTE\times (1-\hbox{exp}\left[-{10}^{3}\times D\right])+{\sum }_{i=LM,H,VH}{kTE}_{i}\times {D}_{i}$	3.0	0.11	0.24	0.18
**NTE_TE**_**_lin_S2**_	$R=B+kNTE\times (1-\hbox{exp}\left[-{10}^{3}\times D\right])+{\sum }_{i=LMH,VH}{kTE}_{i}\times {D}_{i}$	**0.0**	0.11	0.24	0.18
NTE_TE__lin_S3_	$R=B+kNTE\times (1-\hbox{exp}\left[-{10}^{3}\times D\right])+{\sum }_{i=H,VH}{kTE}_{i}\times {D}_{i}$	20.5	0.11	0.24	0.18
NTE_TE__lin_S4_	$R=B+kNTE\times (1-\hbox{exp}\left[-{10}^{3}\times D\right])+{\sum }_{i=VH}{kTE}_{i}\times {D}_{i}$	31.8	0.11	0.24	0.18
NTE_TE__quad_	$R=B+kNTE\times (1-\hbox{exp}\left[-{10}^{3}\times D\right])+{\sum }_{i}{kTE}_{i}\times {D}_{i}^{2}$	7.3	0.11	0.24	0.18
NTE_TE__quad_S1_	$R=B+kNTE\times (1-\hbox{exp}\left[-{10}^{3}\times D\right])+{\sum }_{i=LM,H,VH}{kTE}_{i}\times {D}_{i}^{2}$	8.1	0.11	0.24	0.18
NTE_TE__quad_S2_	$R=B+kNTE\times (1-\hbox{exp}\left[-{10}^{3}\times D\right])+{\sum }_{i=LMH,VH}{kTE}_{i}\times {D}_{i}^{2}$	4.1	0.11	0.24	0.18
NTE_TE__quad_S3_	$R=B+kNTE\times (1-\hbox{exp}\left[-{10}^{3}\times D\right])+{\sum }_{i=H,VH}{kTE}_{i}\times {D}_{i}^{2}$	24.8	0.11	0.24	0.18
NTE_TE__quad_S4_	$R=B+kNTE\times (1-\hbox{exp}\left[-{10}^{3}\times D\right])+{\sum }_{i=VH}{kTE}_{i}\times {D}_{i}^{2}$	39.1	0.11	0.24	0.18

*D* is the radiation dose, *R* is the response variable, *B* is the baseline response parameter, *kTE* is a parameter for targeted effects, *kNTE* is a parameter for non-targeted effects, and *i* is an index that represents the LET category (L = 0.22, M = 13–16, H = 41–50, VH = 106-181 keV/µm). In some simplified models (labeled S1 to S4), TE parameters were allowed to be adjustable only for specified LET categories (*e.g. i* = LM, H, VH indicates that TE parameters were the same for L and M LET categories, but different for H and VH categories) and/or set to zero for certain LET categories (*e.g. i* = VH indicates that TE parameters were non-zero only for the VH LET category). ∆AICc indicates relative information theoretic support for a given model. The best-supported model has ∆AICc = 0 (indicated in bold font), and ∆AICc > 6 suggest poor support (*i.e.* > 20-fold lower, relative to the best model). Coefficient of determination (R^2^), root mean squared error (RMSE) and mean absolute error (MAE) represent absolute goodness of fit metrics.

The structure of the NTE terms in the tested models (Table 1) is based on our previous publications^8,9^. We assume that stress response signals from irradiated cells propagate to other cells and cause them to enter into a stressed “activated” state, which can be persistent. This NTE process is assumed to be binary (“on/off”), so that the probability of the effect (but not its magnitude) increases with dose. In the NTE state, cells can experience oxidative stress, elevated rate of DNA damage, along with other modifications of functioning. The total radiation effect is assumed to be the sum of TE and NTE components.

Based on these assumptions, the commonly observed tendency of NTE dose responses to have a steep initial “rise” at low doses, followed by saturation towards a “plateau” at higher doses, was modeled by the following mathematical expression, where *D* is radiation dose, *kNTE*_*r*_ is the “rise” parameter and *kNTE* is the “plateau” parameter: *kNTE* × (1 – exp[-*kNTE*_*r*_ × *D*]). Preliminary fitting attempts showed that *kNTE*_*r*_ attained a very high value with a very large uncertainty. Consequently, we fixed *kNTE*_*r*_ at 10^3^ Gy^−1^ instead of allowing it to be freely adjustable. In essence, using this large constant allows the response to rapidly increase from the background value and saturate at low doses, but retains the model’s properties as a smooth function instead of a biologically implausible step function.

Each formalism described in Table 1 was fitted to the data. Since the evaluated formalisms are not linear (*e.g.* contain exponential terms), fitting them required nonlinear regression. Initial calculations revealed that the ordinary least squares (OLS) approach in the context of nonlinear regression (implemented by the *nls* function in *R*) violated the assumption of normally-distributed residuals, assessed by the Shapiro–Wilk normality test. For this reason, we used a robust nonlinear regression algorithm (the *nlrob* function in *R*). Robust regression reduces the effects of potential outlier data points. This can be done by using a weighting (loss) function that is different from the commonly used sum of squared errors and assigns less weight to outlier observations^27,28^.

### Assessments of model performance

The performances of different models were compared by information theoretic analysis using the Akaike information criterion with sample size correction (AICc)^29,30^. This approach is useful because it takes into account not only the closeness of model fit to the data (the maximized likelihood value), but also model complexity (the number of adjustable parameters) and the sample size. More complex models are “penalized” more strongly, compared with simpler models, and the penalty increases at small sample sizes. ∆AICc, defined as the given model’s AICc score minus the minimum AICc score across all tested models, indicates relative information theoretic support for a given model. This relative support is quantified by the equation exp[-∆AICc/2]. Therefore, the best-supported model has ∆AICc = 0, and ∆AICc > 6 suggest poor support (*i.e.* > 20-fold lower, relative to the best model). In addition, for each model we calculated the coefficient of determination (R^2^), root mean squared error (RMSE), and mean absolute error (MAE).

To test the stability of the best-supported model variant (the one with ∆AICc = 0) behaviors under random perturbations of the data, we performed 300 random 50:50 splits of the data set into training and testing parts. In each split, the model was fitted to the training data and the predictions from this fit were tested on the testing data. R^2^, RMSE, MAE, and model parameter values were also calculated and compared on the training and testing data.

### Quantile regression

For the best-supported model variant (the one with ∆AICc = 0), we performed quantile regression (using the *quantreg* R package) to model the median (50th percentile), as well as the 25th and 75th percentiles of the radiation response. Quantile regression has a fairly long history of use in biology, medicine and in other fields (e.g.^31,32^). Its important advantages include the ability to handle nonlinear dependences of the outcome on the predictors and non-normal distribution of the outcome. Even more importantly, quantile regression allows not only the mean to be modeled, but also quantifies relationships between predictor variables and several selected percentiles of the data. Therefore, the quantile regression approach provides additional information about model uncertainties and data spread without some of the stringent assumptions of OLS regression and is therefore quite useful for this data set.

### Mixed effects modeling

Since the novel object recognition test was repeated for most rats twice at different time points, correlations of the responses shown by the same rat at different times could be potentially important, but were not accounted for by the robust and quantile regressions. In other words, although time since irradiation apparently did not play an important role in response magnitudes, a given rat could consistently exhibit higher than average (or lower than average) responses whenever it was tested, causing the data points corresponding to this rat not to be statistically independent. To address this issue, we implemented nonlinear mixed effects modeling (*nlme R* package) using the best-supported model variant. This approach is commonly used in situations such as the one here, where repeated measurements are made on the same individuals. In addition to the fixed effects, random effects by rat were included for the model parameters.

To reduce potential outlier data point effects on the mixed effects model fit, we first analyzed the data set by the *OutlierDetection R* package and removed 40 outliers (883 samples were retained). The data set version without these outliers (provided in Supplementary Data File online) was used for the mixed effects modeling, whereas the full data set was used for all other analyses. The Fligner-Killeen test for homogeneity of response variances was significant for radiation dose (*D*) and LET (*p* values 0.00017 and 0.016, respectively), but not for time (*p* value 0.080). Consequently, in the mixed effects model the variance function (*weights* in *nlme*) was allowed to vary by *D* and LET. The following analyses of model residuals were used to diagnose potential violations of the main assumptions: plot the residuals, regress residuals vs *D* and time, boxplot by LET, Shapiro–Wilk normality test, *qq* plot, calculate skewness and kurtosis, and plot the autocorrelation function.

### Machine learning analysis

To assess whether or not the NTE functional form (NTE_f_ = 1 − exp[−10^3^ × *D*]) is important for describing the data outside of the parametric regression framework described above, we performed a machine learning analysis based on random forests (RF). RF is a powerful and frequently utilized machine learning method, which captures complex dependences in the data by generating ensembles of decision trees^33^. The techniques of bootstrap aggregation or “bagging” (randomly selecting samples from training data with replacement) and “feature randomness” (selecting a subset of predictors randomly for each tree) are used in RF to improve performance. For regression problems such as the one considered here, predictions from all trees are averaged.

For the machine learning analysis, we considered radiation dose (*D*), energy (in MeV/n), time since exposure, LET (in keV/µm), and NTE_f_ as predictors for the response. The data set was split randomly into training and testing halves. The Boruta feature selection algorithm^34^ (*Boruta* package in *R*) was implemented on the training data to generate a ranking of importance scores for the predictor variables. Boruta iteratively compares the RF-based importance score of each predictor with the importance score of its randomly shuffled “shadow”. The Boruta analysis was repeated 100 times with different initial random number seeds, and the predictor variables were ranked by the median value of median importance scores across all repeats. This procedure was intended to identify the most important predictors and/or the least important ones, which could potentially be discarded from further analysis.

Using the retained important predictors, we implemented RF (2000 trees), optimizing its parameters (number of variables to possibly split at in each node *mtry*, splitting rule *splitrule*, and minimal node size *min.node.size*) by RMSE using repeated cross-validation (threefold, 30 repeats) on the randomly-selected training half of the data. Performance (R^2^, RMSE and MAE) was measured on the testing half of the data. This approach, designed to minimize the probability of “overfitting”, was implemented by the *caret* and *ranger R* packages. Robustness of RF predictions and performance metrics to random data fluctuations was assessed by applying the algorithm (with previously optimized parameters) to 300 random training/testing splits of the original data set.

## Results

The NTE_TE__lin_S2_ formalism outperformed all other models based on AICc scores (Table 1). This formalism assumes that NTE saturate at low doses (~ 0.01 Gy) and occur at all tested LETs, whereas TE depend on dose linearly with a slope that increases with LET. The best-supported model’s parameters and performance metrics are provided in Table 2. These parameter values remained relatively stable when the model was fitted to randomly-selected training parts of the data set and tested on the corresponding testing parts (Table 2).

**Table 2:** Performance of the best-supported NTE_TE__lin_S2_ model (Table 1).

Parameter or metric	Value for full data set (SE, *p* value)	Mean value on training data (SD) [range]	Mean value on testing data (SD) [range]
*B*	0.424 (0.020, < 2 × 10^−16^)	0.424 (0.011) [0.393, 0.455]	-
*kNTE*	0.166 (0.025, 6.7 × 10^−11^)	0.166 (0.017) [0.125, 0.204]	-
*kTE*_*i*=*LMH*_	0.027 (0.017, 0.12)	0.026 (0.016) [0, 0.077]	-
*kTE*_*i*=*VH*_	0.127 (0.035, 2.6 × 10^−4^)	0.126 (0.031) [0.023, 0.233]	-
R^2^	0.109	0.112 (0.016) [0.068, 0.157]	0.104 (0.016) [0.067, 0.148]
RMSE	0.242	0.240 (0.007) [0.218, 0.260]	0.243 (0.007) [0.223, 0.262]
MAE	0.183	0.182 (0.004) [0.166, 0.194]	0.184 (0.004) [0.173, 0.200]

As described in Materials and Methods, the full data set was randomly split 300 times into training and testing halves. The model fits to training data were used to calculate R^2^, RMSE and MAE metrics on both training and testing data. SE = standard error, SD = standard deviation.

Three other model variants had support values close to the best model (ΔAICc < 6): NTE_TE__lin_S1_, NTE_TE__lin_, and NTE_TE__quad_S2_ (Table 1). The first two of these models assume more detail for the linear TE slope variation by LET categories, and the last one assumes a quadratic TE dose response (Table 1). Other performance metrics besides AICc (R^2^, RMSE, MAE) were very similar for these models and for the best model (Table 1). These results suggest that the data set and analysis methods do not provide strong confidence in the details of TE dependences on dose and LET. However, they do provide a strong indication that the NTE model terms are important because these terms are present in all model variants with the best support (ΔAICc < 6).

**Figure 1:**
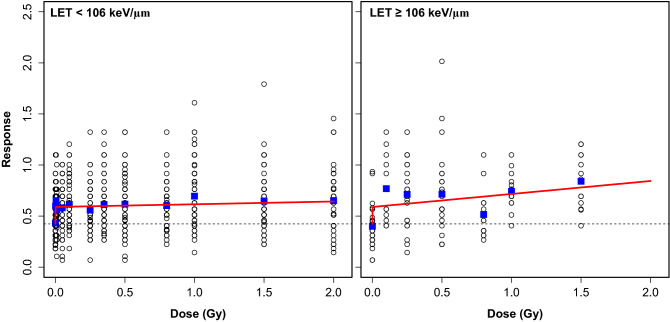
Comparisons of best-fit predictions (red curves) for the best-supported NTE_TE__lin_S2_ model (Table 1) with the data (black circles). Blue squares indicate mean response values at each dose, and the black dashed line indicates baseline response in unirradiated rats. As described in the main text, the response variable is defined as the negative logarithm of the fraction of time spent exploring a novel object. This implies that higher response values indicate more severe radiation damage to novel object recognition. Our model assumes that the TE dose response “slope” differs by LET category, whereas NTE occur at all LET values.

**Figure 2:**
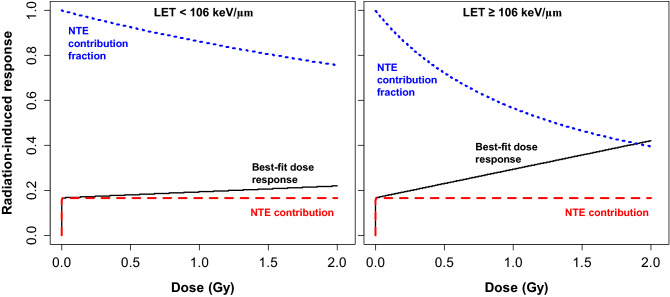
Visualization of the NTE contribution (absolute, red dashed curves, and fractional, blue dashed curves) to the dose response predictions (black curves) of the best-supported NTE_TE__lin_S2_ model (Table 1). Baseline response in unirradiated rats (parameter *B*) was subtracted for improved visualization.

Visualizations of the best-supported NTE_TE__lin_S2_ model’s predictions, compared with the data points, are shown in Fig. 1. The contributions of the TE and NTE components to the predicted radiation response are shown in Fig. 2. Based on this model, NTE dominate at low radiation doses (*e.g.* < 0.5 Gy), whereas TE dominate only at high doses of very high LET (≥ 106 keV/µm) radiation (Fig. 2).

Quantile regression using the best-supported NTE_TE__lin_S2_ model showed that the TE parameter for LET values < 106 keV/µm had large uncertainties (including zero) across quantiles (25th and 75th percentiles). In contrast, the NTE parameter and the TE parameter for LET values ≥ 106 keV/µm were relatively stable (Table 3).

**Table 3:** Parameters for quantile regression using the best-supported NTE_TE__lin_S2_ model (Table 1).

Parameter	Best-fit value (SE, *p* value)		
	50th percentile	25th percentile	75th percentile
*B*	0.424 (0.015, < 10^−6^)	0.310 (0.013, < 10^−6^)	0.511 (0.025, < 10^−6^)
*kNTE*	0.166 (0.030, < 10^−6^)	0.147 (0.021, < 10^−6^)	0.220 (0.037, < 10^−6^)
*kTE*_*i*=*LMH*_	0.027 (0.023, 0.25)	0 (0.020, 1.0)	0.053 (0.031, 0.093)
*kTE*_*i*=*VH*_	0.127 (0.045, 5.1 × 10^−3^)	0.074 (0.047, 0.12)	0.182 (0.064, 4.7 × 10^−3^)

*SE* standard error.

Mixed effects modeling provided support for random effects (*i.e.* variability of parameter values by rat) only for baseline responses in non-irradiated rats (parameter *B*). Adding random effects for the NTE and/or TE parameters resulted in poor convergence and/or negligibly small standard deviations (< 10^−3^) for the random effects. The mixed effects model variant with random effects for *B* only had the following parameter values. Fixed effects: *B* = 0.432 (standard error, SE: 0.014, *p* value: < 10^−5^), NTE = 0.165 (SE: 0.019, *p* value: < 10^−5^), TE for LET values < 106 keV/µm = 0.032 (SE: 0.015, *p* value: 0.028), TE for LET ≥ 106 keV/µm = 0.060 (SE: 0.046, *p* value: 0.20). The random effects standard deviation for *B* was 0.073, suggesting substantial variation in baseline responses among rats. These mixed effect model parameter values (especially NTE terms) are generally similar to those produced by the robust and quantile regressions (Tables 2, 3). Most of the variation between rats appeared to occur in baseline responses, not in radiation effects. However, we note that the mixed effect model residuals violated the normality assumption (Shapiro–Wilk test *p* value 4.3 × 10^−9^, skewness 0.53, kurtosis 3.40), so the parameter estimates from this model may have limited reliability.

Machine learning analysis by the Boruta feature selection algorithm calculated the following median importance scores for the considered predictors: NTE term (NTE_f_) = 15.2, radiation dose (*D*) = 14.6, ion energy = 9.0, LET = 8.9, time since exposure = 6.9. All of these variables outperformed randomized “shadow” features > 90% of the time, so all were retained for further analysis by random forest (RF). RF with optimized parameters also ranked NTE_f_ and *D* as the two most important predictors with relative importance scores of 100.0 and 69.4, respectively. Due to its flexibility in describing nonlinear relationships and interactions between predictors by tree ensembles, RF achieved higher performance metrics than the parametric models: mean R^2^ on testing data over 300 random training/testing splits was 0.19 (standard deviation, SD = 0.02, range: 0.13–0.29), RMSE was 0.23 (SD = 0.007, range: 0.21–0.25), MAE was 0.17 (SD = 0.004, range: 0.16–0.18).

## Discussion

We analyzed a large data set on novel object recognition (NOR) testing in rats exposed to a wide range of space radiation types and doses^26^ using several dose response model variants with TE or NTE terms, and several techniques with different underlying assumptions. The NOR task is a measure of the ability of the subject to recall an interaction with a specific object. Normal (unirradiated) rats will spend significantly more time with a novel object than with a familiar object. Subjects with impaired memory spend equal amounts of time with both the novel and familiar objects. As such, novel object performance is a measure of memory.

The model formalisms used here are based on the framework developed in our previous publications^8,9^. This approach allows for assessment of distance for NTE signal propagation^8^, but in situations relevant for space exploration missions, such as the one here, radiation exposure was assumed to be homogeneous throughout the target organ/organism on a macroscopic scale. Consequently, the concentration of NTE signals throughout the organ/organism was also assumed to be homogeneous, and the NTE dose response in such situations depends on the probability of cells to enter and stay in a stressed state. This set of assumptions is consistent with a dose response function with a steep initial “rise” at low doses, where the probability of NTE signal release and response to these signals increases, followed by saturation towards a “plateau” at higher doses, where most susceptible cells respond to the signals. Conceptually similar approaches were also used by other authors^11,35,36^.

Our results show that models with NTE terms described the data much better than those with only TE terms (Table 1). The detailed structure of the NTE dependence on dose and LET could not be determined using this data set and analysis methods, but the importance of including NTE terms (in addition to TE terms) for describing this data set is clear based on the results. The finding that NTE may saturate at very low doses is not unique to this data set, but is consistent with other studies^9,17,36^. Biologically, it suggests that signals released from a cell heavily damaged by radiation can affect large numbers of surrounding cells. This cell “group” response to radiation damage caused in only a small fraction of group members can explain a very steep dose response at very low doses. The specific mechanisms remain to be elucidated, but recent data suggest that microglia activation and neuroinflammation may be involved^37,38^.

In summary, our analysis suggests that radiation effects on novel object recognition can be induced even at low doses of space radiations, and that the dose response in this space-relevant dose range is not linear (concave) and is likely dominated by NTE rather than TE. Importantly, the radiation effects were persistent and not significantly affected by time since exposure, which spanned a substantial portion of the rat lifetime. This provides evidence that radiation-induced cognitive decline may not just occur during a space exploration mission, but can potentially last over a lifetime. Our findings are of course based on a single (although large) data set in laboratory animals. However, we believe that they have potentially important implications for assessing CNS dysfunction risks for astronauts on interplanetary space missions.

## Supplementary Information

Supplementary Information
